# Supplementary material for: AgRP neuron cis-regulatory analysis across hunger states reveals that IRF3 mediates leptin’s acute effects
Source: Nat Commun. 2024 May 31;15:4646. doi: 10.1038/s41467-024-48885-y (PMC11143326; doi:10.1038/s41467-024-48885-y)
Supplement: Supplementary file 1 — Supplementary Info [file 41467_2024_48885_MOESM1_ESM.pdf]

AgRP neuron cis-regulatory analysis across hunger states reveals that IRF3 mediates leptin's acute effects

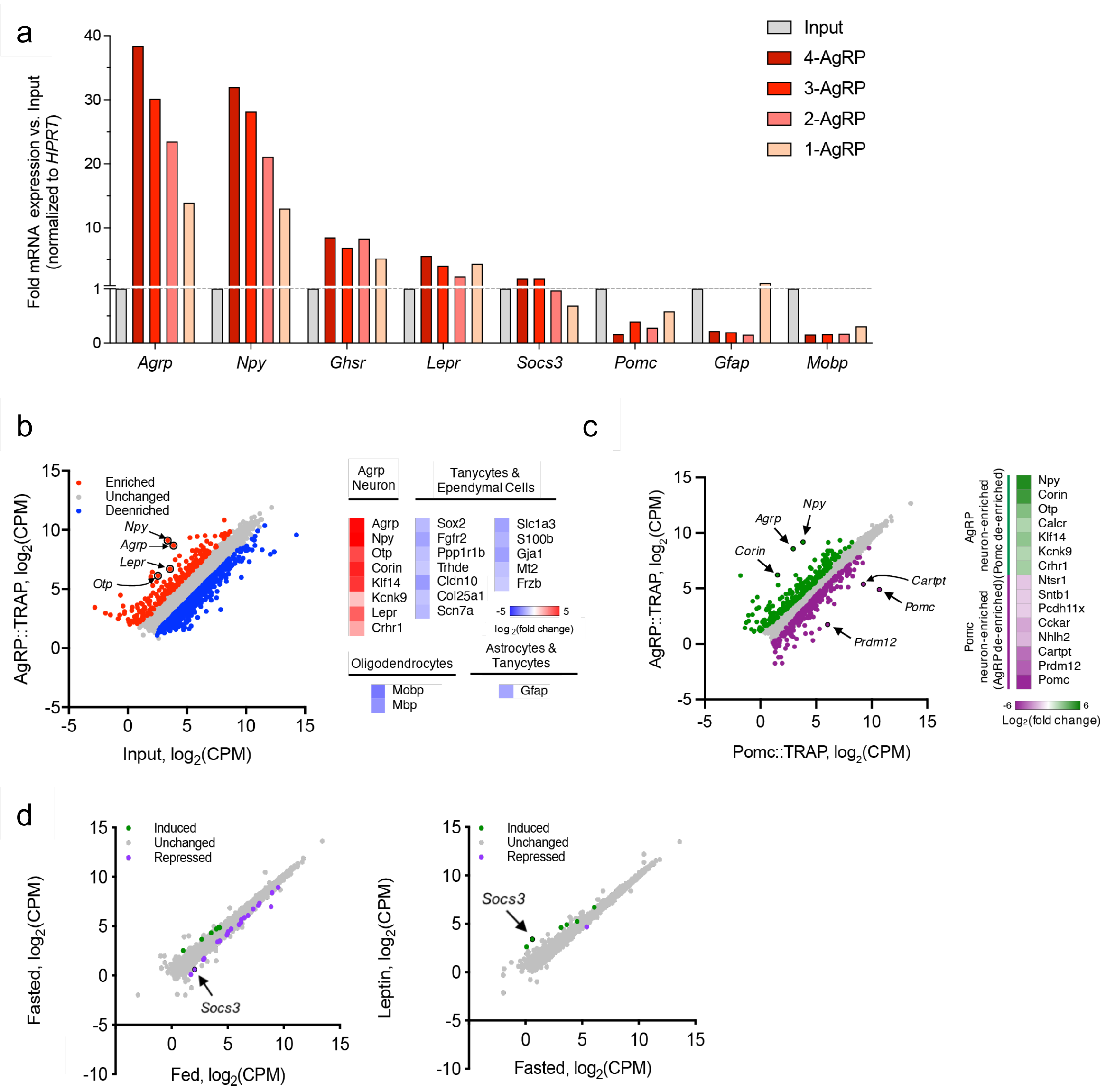

Supplementary Figure 1. Establishment of TRAP-seq for AgRP neurons. (a) Gene expression analysis by qPCR with TRAP-isolated RNA from a titration of 4-to-1 pooled NuTRAP^{AgRP} ARCs. Bars indicate the fold mRNA expression vs. input for each sample. **(b)** Scatter plot of RNA-seq data showing regulated genes from TRAP-enriched AgRP neurons versus input control fraction of these same samples (*n* = 3, 3) [fold change > 0.5 up (red) or down (blue) and false discovery rate (FDR) < 0.05]; CPM, counts per million. The number of genes in a given expression profile is in parentheses. **(c)** Scatter plot of RNA-seq data showing regulated genes within AgRP neurons from TRAP-enriched AgRP neurons versus Pomc neurons from TRAP-enriched Pomc neurons (*n* = 3, 2) [fold change > 0.5 up (red) or down (blue) and false discovery rate (FDR) < 0.05]; CPM, counts per million. **(d)** Left: Scatter plot of RNA-seq data showing regulated genes within Pomc neurons from fasted versus fed littermates (n = 2, 2) [fold change >0.5 up (red) or down (blue) and false discovery rate (FDR) < 0.05]; CPM, counts per million. Right: Scatter plot of RNA-seq data showing regulated genes within Pomc neurons from leptin-treated versus fasted littermates (n = 2, 2) [fold change >0.5 up (red) or down (blue) and false discovery rate (FDR) < 0.05]; CPM, counts per million.

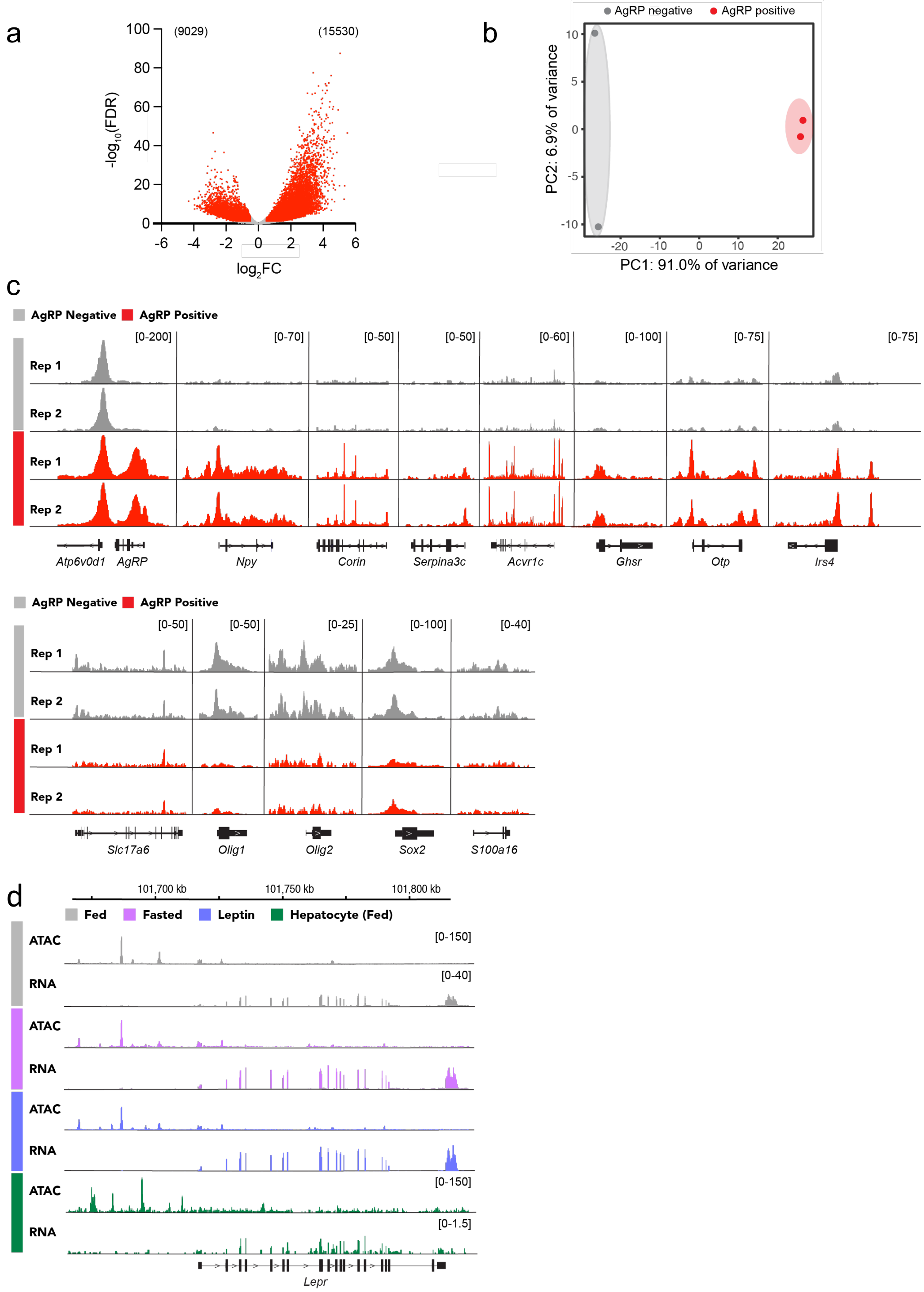

Supplementary Figure 2. ATAC-seq profiles of AgRP neurons. (**a**) Volcano plot showing differential OCRs upon comparing AgRP positive vs. AgRP negative (n = 2, 2) neurons. Red dots correspond to significantly different gained-open and gained-closed regions [fold change >0.5 up (red) or down (red) and false discovery rate (FDR) < 0.05]; CPM, counts per million. Peaks with a CPM < 1 were not included in volcano plot. (**b**) Principal Components Analysis (PCA) plot comparing AgRP negative and AgRP positive ATAC-seq samples. (**c**) Genome browser views (IGV) of representative ATAC-seq peaks near genes enriched (top) and de-enriched (bottom) in AgRP neurons. (**d**) Genome browser views (IGV) of *Lepr*-associated ATAC-seq and RNA-seq peaks in the fed, fasted, and leptin-treated states.

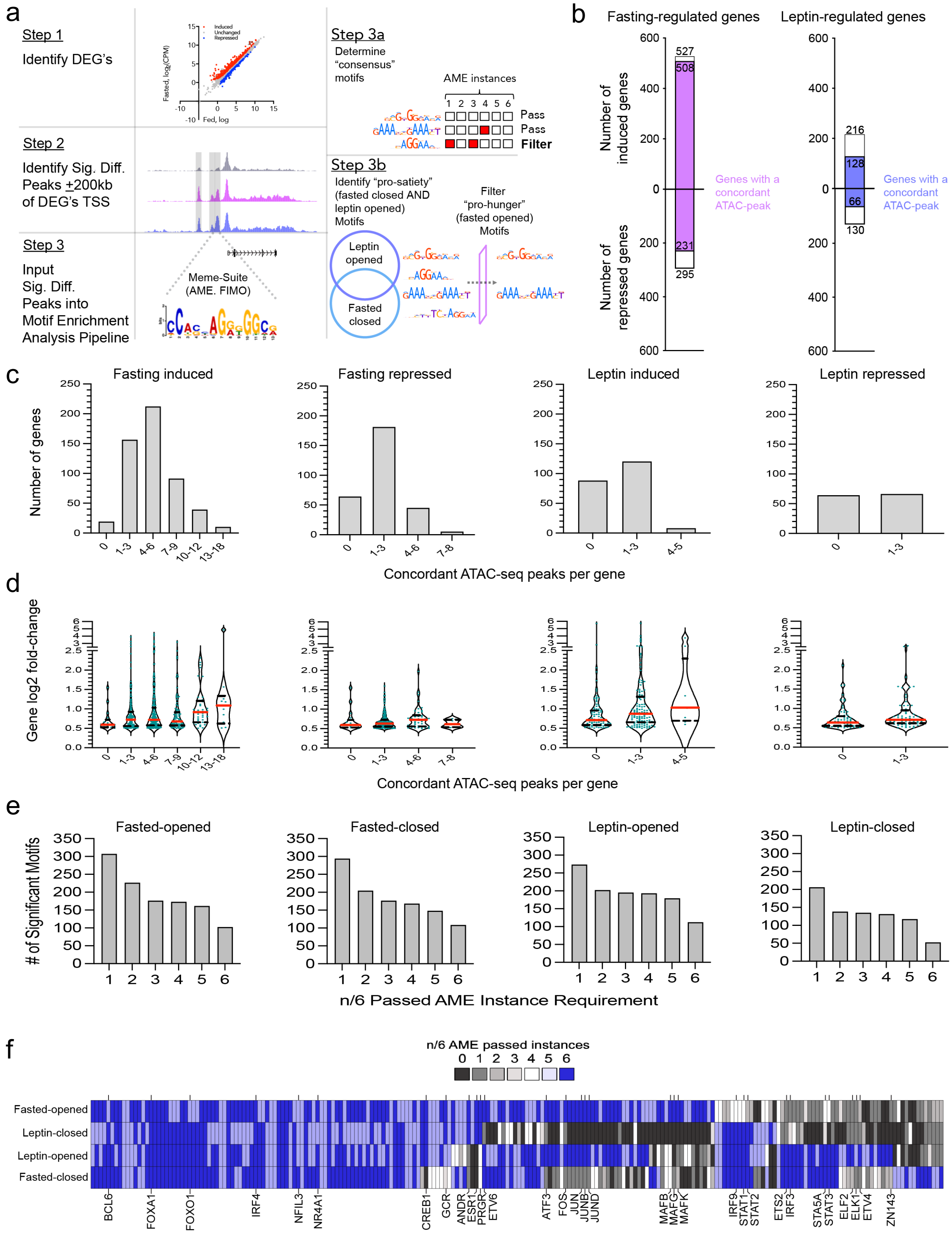

Supplementary Figure 3. **Gene expression and chromatin state metrics of AgRP neurons.** (**a**) Schematic showing the detailed computational heuristic employed to identify putative pro-satiety TF motifs. Step 1: Identify fasted- and leptin-induced differentially expressed genes (DEGs). Step 2: Determine significantly different fasted-opened, fasted-closed and leptin-opened ATAC-seq peaks, and identify those that are ± 200 kb upstream and downstream of those DEGs identified in Step 1 (e.g., gene-linked ATAC-seq peaks). Step 3a: Perform motif enrichment analysis on the peaks identified in Step 2. Step 3b: Determine the consensus motif

identified in 5 out of 6 instances of analysis of motif enrichment (AME). Step 3c: Identify putative pro-satiety TF motifs that are dually enriched in leptin-opened and fasted-closed peaks, but not fasted-opened (putatively pro-hunger) peaks. **(b)** Left: bar graphs showing number of fasted-induced and fasted-repressed genes, with internal bar showing the number of those genes that have a concordant ATAC peak. Right: bar graphs showing number of leptin-induced and leptin-repressed genes, with internal bar showing the number of those genes that have a concordant ATAC peak. Genes without an annotated TSS have no concordant peaks under any condition (*p*-values: 6.767e-13 and 1.99e-11 by Fisher's exact test for concordant changes with fasting and leptin, respectively). **(c)** Number of genes that possess a given number range of concordant ATAC-seq peaks per gene for fasting-induced, fasting-repressed, leptin-induced, leptin-repressed conditions. Distributions are significantly shifted rightwards for fasting- and leptin-induced concordant peak-gene pairs (*p*-values: 1.55e-14 and 1.312e-07 via one-sided Mann-Whitney test). (**d**) Mean gene fold-change (red bar) for binned numbers of concordant ATAC-seq peaks per gene for fasting-induced, fasting-repressed, leptin-induced, leptin-repressed conditions. The violin plots indicate median values (red line), first and third quartiles (dashed lines). (**e**) Number of significant motifs for each of the passed AME instance requirements (1-6), for fasted-opened, fasted-closed, leptin-opened, and leptin-closed conditions. (**f**) Heatmap of the number of AME instances that passed the significance threshold (i.e., n $\geq$ 5 out of 6 AME instances).

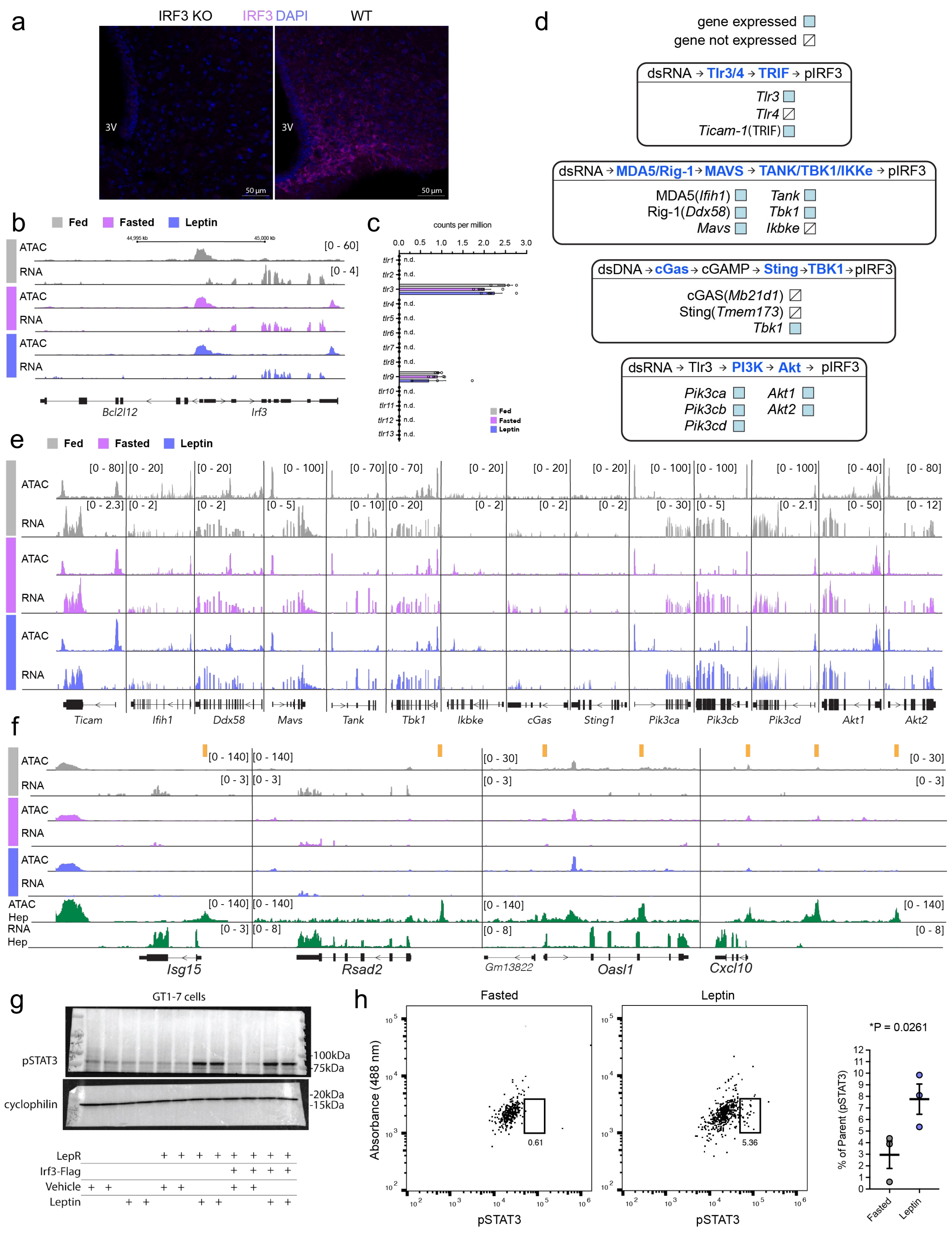

Supplementary Figure 4. Establishment of a system for studying activation of IRF3 by leptin signaling. (a) Immunofluorescence for IRF3 (magenta), with DAPI (blue), in the mouse ARC in the fed state of a WT mouse (right) and global IRF3 knockout mouse (left); repeated independently once. **(b)** ATAC-seq and RNA-seq reads at the *Irf3* locus under fed, fasted, and leptin-treated conditions. **(c)** mRNA expression from NuTRAP^{AgRP} TRAP-seq experiment, showing the expression of all 13 toll-like receptors. Gene not detected (n.d.). Each circle denotes a single pooled sample. Results are mean ± SEM of each condition. These results originated from the Figure 1 TRAP-seq experiment, thus they were not reanalyzed, while being included for illustrative purposes. **(d)** Relevant pathways for IRF3 activation, with blue boxes indicating the expression of

the gene in our AgRP neuron RNA-seq dataset. **(e)** Genome browser views (IGV) showing AgRP neuron RNA-seq and ATAC-seq tracks for genes associated with IRF3 activation. **(f)** Genome browser views (IGV) showing AgRP neuron and hepatocyte RNA-seq and ATAC-seq tracks for genes canonically associated with IRF3 activation. Yellow boxes delineate ATAC-seq peak positions that are dissimilar between AgRP neurons and hepatocytes. (**g**) Western blot showing the leptin-responsivity of transfected GT1-7 cells treated with leptin. (**h**) Two representative scatter plots of pSTAT3-Alexa Fluor647 primary and secondary staining (x-axis) and empty 488 channel (y-axis) were used to identify the pSTAT3-positive population. Flow cytometry data were generated from three independent biological replicates for fasted mice, and three independent biological replicates for leptin-treated mice. Results are mean ± SEM of each condition and analyzed by a one-tailed student t-test ($p = 0.0261$, $t=2.735$, $df=4$).

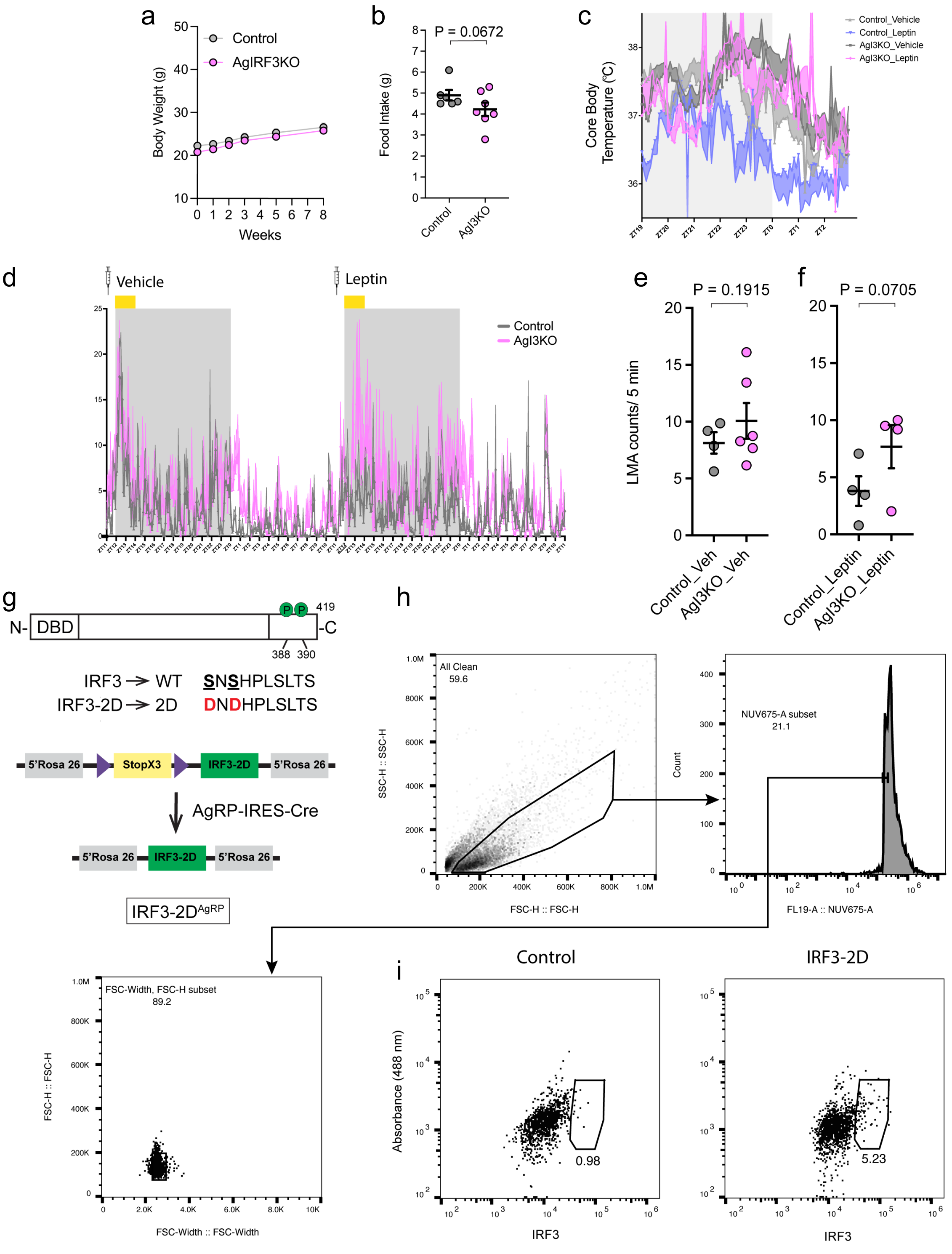

Supplementary Figure 5. IRF3 loss-of-function and gain-of-function models in AgRP neurons. Associated with main Figure 5. (a) Body weight of chow-fed control and AgI3KO mice over an 8-week period. **(b)** 24-hour food-intake of chow-fed control and AgI3KO mice. Each circle denotes a single mouse. Data in Figures 5A and 5B were generated from six independent biological replicates for control mice, and seven independent biological replicates for AgI3KO mice. Results are mean ± SEM of each condition and analyzed by a one-tailed student t-test ($p = 0.0672$, $t=1.616$, $df=11$). (**c**) Inset image showing superimposed Control_Vehicle, Control_Leptin, AgI3KO_Vehicle, and AgI3KO_Leptin core body temperature plots during their respective second 8-hour recording epochs. **(d)** Locomotor activity (LMA) measurements during a 48-hour period encompassing the vehicle and leptin injected trials involving control or AgI3KO mice, with syringes and

yellow boxes illustrating the times of vehicle/leptin injection and the 2-hour epochs used for analyses, respectively. **(e)** 2-hour LMA levels comparing vehicle-treated control vs. AgI3KO experiment (n = 4, 6), and (**f**) the leptin-treated control vs. AgI3KO experiment (n = 4, 4). Locomotion data in Figure S5E were generated from four independent biological replicates for control mice, and six independent biological replicates for AgI3KO mice. Locomotion data in Figures S5F were generated from four independent biological replicates for control mice, and four independent biological replicates for AgI3KO mice. Each circle depicts a mouse's average mean LMA value across each 5-minute bin. (**e**) Results are mean ± SEM of each condition and analyzed by a one-tailed student t-test (p = 0.1915, t=0.9232, df=8). (**f**) Results are mean ± SEM of each condition and analyzed by a one-tailed student t-test (p = 0.0705, t=1.695, df=6). **(g)** Schematic of the AgI3-2D mouse transgene. (**h**) Flow cytometry gating strategy. Particles smaller than nuclei (black dots) were eliminated with an area plot of forward-scatter (FSC-A) versus side-scatter (SSC-A), with gating for nuclei-sized particles inside the gate (box). 2N Hoechst 33342 stained nuclei were positively gated to avoid nuclei doublets. Plots of forward-scatter (FSC) width versus FSC height were used to further exclude aggregates of two or more nuclei. (**i**) Two representative scatter plots of IRF3-Alexa Fluor647 primary and secondary staining (x-axis) and empty 488 channel (y-axis) were used to identify the IRF3-positive population. Numbers reflect the % of the parent population.

Supplementary Table 1. qRT-PCR primers.

Gene	Forward	Reverse
Agrp	TGCTGAGTTGTGTTCTGCTG	GTCTTCTTGAGGCCATTCAGAC
Npy	CAGCCCTGAGACACTGATTTC	ACATGGAAGGGTCTTCAAGC
Ghsr	TCTTCTGCCTCACTGTGCTC	CAGGCTCGAAAGACTTGGAA
Lepr	GCAGTGTACTGCTGCAATGAG	ATTGGATTGTGCTGGGTGAC
Socs3	AGACCTTCAGCTCCAAAAGC	TGTCGCGGATAAGAAAGGTG
Pomc	CATAGATGTGTGGAGCTGGTG	CAGCGAGAGGTCGAGTTTG
Gfap	TCGCCACCTACAGGAAATTG	GGATCTGGAGGTTGGAGAAAG
Mobp	CAAGAGCGGTTGCTTTTACC	TCACTTCTTCCTTGGGGTTG
