# Supplementary material for: AgRP neuron cis-regulatory analysis across hunger states reveals that IRF3 mediates leptin’s acute effects
Source: Nat Commun. 2024 May 31;15:4646. doi: 10.1038/s41467-024-48885-y (PMC11143326; doi:10.1038/s41467-024-48885-y)
Supplement: Supplementary file 3 — Description of Supplementary Information [file 41467_2024_48885_MOESM3_ESM.pdf]

Description of Supplementary Files for
“AgRP neuron cis-regulatory analysis across hunger states reveals that IRF3 mediates leptin’s acute effects”

File Name: **Supplementary Dataset 1**
Description: Gene enriched and de-enriched in AgRP neurons.

File Name: **Supplementary Dataset 2**
Description: AgRP neuron Fed Fasted and Leptin TRAP-seq results.
