# Supplementary material for: AgRP neuron cis-regulatory analysis across hunger states reveals that IRF3 mediates leptin’s acute effects
Source: Nat Commun. 2024 May 31;15:4646. doi: 10.1038/s41467-024-48885-y (PMC11143326; doi:10.1038/s41467-024-48885-y)
Supplement: Supplementary file 5 — Reporting Summary [file 41467_2024_48885_MOESM5_ESM.pdf]

nature portfolio

Corresponding author(s): Frankie D. Heyward; Evan D. Rosen

Last updated by author(s): Apr 16, 2024

Reporting Summary

Nature Portfolio wishes to improve the reproducibility of the work that we publish. This form provides structure for consistency and transparency in reporting. For further information on Nature Portfolio policies, see our Editorial Policies and the Editorial Policy Checklist.

Statistics

For all statistical analyses, confirm that the following items are present in the figure legend, table legend, main text, or Methods section.

n/a	Confirmed	
☐	☒	The exact sample size (*n*) for each experimental group/condition, given as a discrete number and unit of measurement
☐	☒	A statement on whether measurements were taken from distinct samples or whether the same sample was measured repeatedly
☐	☒	The statistical test(s) used AND whether they are one- or two-sided<br>*Only common tests should be described solely by name; describe more complex techniques in the Methods section.*
☒	☐	A description of all covariates tested
☐	☒	A description of any assumptions or corrections, such as tests of normality and adjustment for multiple comparisons
☐	☒	A full description of the statistical parameters including central tendency (e.g. means) or other basic estimates (e.g. regression coefficient) AND variation (e.g. standard deviation) or associated estimates of uncertainty (e.g. confidence intervals)
☐	☒	For null hypothesis testing, the test statistic (e.g. *F*, *t*, *r*) with confidence intervals, effect sizes, degrees of freedom and *P* value noted<br>*Give P values as exact values whenever suitable.*
☒	☐	For Bayesian analysis, information on the choice of priors and Markov chain Monte Carlo settings
☒	☐	For hierarchical and complex designs, identification of the appropriate level for tests and full reporting of outcomes
☒	☐	Estimates of effect sizes (e.g. Cohen's *d*, Pearson's *r*), indicating how they were calculated

Our web collection on statistics for biologists contains articles on many of the points above.

Software and code

Policy information about availability of computer code

Data collection

No software was used to collect the data in this study.

Data analysis

RNA-seq Analysis
RNA-seq data was aligned against the mm10 genome using HISAT2 (Kim et al., 2015). Primary alignments to exons were counted and summarized at the gene level by thge featureCounts program (Liao et al., 2014), part of the Subread software package. Differential expression analysis of the data was performed using edgeR (Robinson et al., 2010). Significantly different genes were required to have an average expression > 1 cpm, a fold change (FC) > 0.5, and an false discovery rate of < 0.05. Gene set enrichment analysis (GSEA) gene ontology (GO) 24 for biological process was carried out with WebGestalt (WEB-based GEne SeT AnaLysis Toolkit) (Wang et al., 2017). Genes with cpm < 1 were omitted from the GSEA analysis.

ATAC-seq data processing
Library reads were aligned to the mm10 (mouse) genome assembly using Bowtie2. Duplicate alignments were filtered out with SAMtools (Li et al., 2009). Peaks were called using MACS2. Peaks were merged and coverage calculated per-sample using BEDtools (Quinlan and Hall, 2010). Differential binding analysis including normalization and quantification were done in edgeR. Differential peaks were defined at log2 fold change > 0.5, a false discovery rate of < 0.05, and an average expression > 1 cpm. ATAC-seq peaks were visualized in the Integrated Genome Browser (IGV).

Motif Enrichment Analysis and TF Prioritization
Transcriptionally concordant ATAC-seq peaks were subjected to Motif Enrichment was determined using the MEME-suite Analysis of Motif Enrichment (AME) (Version 5.1.0) sequence analysis tool (Figure 3A)(Bailey et al., 2015). We restricted our analysis to significant concordant peaks for each of the four significant ATAC-seq peak patterns (i.e., fasted-opened, fasted-closed, leptin-opened, leptin-closed). AME was

conducted 6 distinct times using one of the 6 possible background control sets of peaks during each analysis: (1) neutral (i.e., unchanging) peaks anywhere in the genome; (2) neutral peaks near neutral genes; (3) neutral peaks near significant genes; (4) neutral peaks near any TSS; (5) all non-concordant ATACseq peaks for a given conditions (e.g., leptin-opened); or (6) concordant ATAC-seq peaks that are shuffled (e.g., leptin-opened peak sequences that are shuffled). Thus, in all, 6 distinct instances of AME were performed for each peak set. We next employed a rule whereby we further considered motifs only if they were significantly enriched in ≥5 out of 6 AME instances (Figure S3A, S3E). To determine the representative log transformed p-values (for plotting purposes), for those "enriched" motifs (at least 5 out of 6 AME instances), the minimal (most significant) p-value out of the 6 motif instances was determined, while omitting from the analysis any non-significant pvalues, for those Motifs that have a single non-significant motif but are retained. Conversely, to determine the representative p-value for those non-enriched motifs (violate the at least 5 out of 6 significant rule) we calculated the minimal (most significant) p-value, of the non-significant ones. We then log transformed the resulting p values (log2). For the transcriptionally concordant fasted-opened condition only, p-values for 12 of the 356 TF motifs were less than e^-300 and thus could not be log transformed (ATF3.0.A, BACH1.0.C, BACH2.0.A, BATF.0.A, CRX.0.A, FOS.0.A, FOSB.0.A, FOSL1.0.A, FOSL2.0.A, JUN.0.A, JUNB.0.A, JUND.0.A). Therefore for these motifs only in fasted-opened condition, the log transformed p-value was determined by imputation. Evidently, none of these 12 TF motifs were qualified (due to enrichment in disqualifying chromatin conditions) to be plotted in figures 3G or 3I. All plots were made using Graphpad PRISM software (Version 9).

samtools ref: https://academic.oup.com/bioinformatics/article/25/16/2078/204688
bedtools ref: https://academic.oup.com/bioinformatics/article/26/6/841/244688
fastp ref: https://academic.oup.com/bioinformatics/article/34/17/i884/5093234?login=false
meme ref: https://www.nature.com/articles/nprot.2014.083

Live Cell Imaging, Fluorescence Intensity Analysis
Mean Fluorescence Intensity of each targeted cell's cytoplasm and nuclei was determined using ImageJ software (NIH) to enable calculation of the nuclear-to-cytoplasm (N/C) GFP intensity ratio.

All scripts used in the current study are available on Zenodo (DOI: 10.5281/zenodo.10967242), the used packages are listed in the Methods session.

For manuscripts utilizing custom algorithms or software that are central to the research but not yet described in published literature, software must be made available to editors and reviewers. We strongly encourage code deposition in a community repository (e.g. GitHub). See the Nature Portfolio guidelines for submitting code & software for further information.

Data

Policy information about availability of data

All manuscripts must include a data availability statement. This statement should provide the following information, where applicable:

- Accession codes, unique identifiers, or web links for publicly available datasets
- A description of any restrictions on data availability
- For clinical datasets or third party data, please ensure that the statement adheres to our policy

All raw and processed RNA-Seq, and ATAC-seq have been deposited in the NCBI Gene Expression Omnibus and have been assigned an accession number GEO: GSE240484.
All scripts used in the current study are available on Zenodo (DOI: 10.5281/zenodo.10967242), the used packages are listed in the Methods session.

Research involving human participants, their data, or biological material

Policy information about studies with human participants or human data. See also policy information about sex, gender (identity/presentation), and sexual orientation and race, ethnicity and racism.

Reporting on sex and gender	NA.
Reporting on race, ethnicity, or other socially relevant groupings	NA.
Population characteristics	NA.
Recruitment	NA.
Ethics oversight	NA.

Note that full information on the approval of the study protocol must also be provided in the manuscript.

Field-specific reporting

Please select the one below that is the best fit for your research. If you are not sure, read the appropriate sections before making your selection.

☒ Life sciences ☐ Behavioural & social sciences ☐ Ecological, evolutionary & environmental sciences

For a reference copy of the document with all sections, see nature.com/documents/nr-reporting-summary-flat.pdf

Life sciences study design

All studies must disclose on these points even when the disclosure is negative.

Sample size	We do not include a justification of sample size for this study. We used the minimum number of animals needed to reliably detect the expected effect size with an alpha rate set at .05 in a standardly powered experiment and based on extensive laboratory experience and literatures in the field.
Data exclusions	2 mice were excluded from the experiment related to Figures 5E, 5G, 5SC, 5SD, 5SF due to mishandling during cannula leptin injection. For RNA-seq a pooled Fed sample (02) was lost during library preparation.
Replication	For RNA-seq experiment three biological replicates, each with 4 pooled mouse ARCs, were used for fed condition, whereas four biological replicates were used for the fasted and leptin-treated conditions. For ATAC-seq experiment two biological replicates, each with 4 pooled mouse ARCs, were used for all conditions. The live-cell imaging experimental result recapitulated an earlier observation from a pilot experiment. The leptin IRF3 flow cytometry finding was replicated once. All behavioral experiments were conducted a single time as reported.
Randomization	For RNA-seq and ATAC-seq experiments, littermates were randomly assigned to a particular group (i.e., Fed, Fasted, Leptin-treated). For food intake experiments all mice were treated with either leptin on the same day, followed by vehicle on a subsequent day, thus obviating the need for randomized group assignment.
Blinding	For food intake experiments, the experimenter who designed the experiment, conducted the experiment. Yet, during the execution of the experiment, the experimenter was unaware of the genotype of the mice during data collection. The experimenter was not blinded to experimental condition during live cell imaging analysis. RNA-seq and ATAC-seq were not performed blind so as to ensure the proper condition designation of all groups. Telemetry experiment data collection were automated, and thus obviated the need for experimental blinding. For live-cell imaging experiment, the experimenter could not be blinded as it was necessary to administer the vehicle or leptin into the proper wells.

Reporting for specific materials, systems and methods

We require information from authors about some types of materials, experimental systems and methods used in many studies. Here, indicate whether each material, system or method listed is relevant to your study. If you are not sure if a list item applies to your research, read the appropriate section before selecting a response.

Materials & experimental systems

n/a	Involved in the study	
☐	☒	Antibodies
☐	☒	Eukaryotic cell lines
☒	☐	Palaeontology and archaeology
☐	☒	Animals and other organisms
☒	☐	Clinical data
☒	☐	Dual use research of concern
☒	☐	Plants

Methods

n/a	Involved in the study	
☒	☐	ChIP-seq
☐	☒	Flow cytometry
☒	☐	MRI-based neuroimaging

Antibodies

Antibodies used	Rabbit Monoclonal IRF3 (used for flow cytometry and western blot), Cell Signaling Technology, Cat# 4302S;<br>Rabbit Monoclonal Phospho-Stat3 (Tyr705) (D3A7) XP (used for flow cytometry and western blot), Cell Signaling Technology, Cat# 9145;<br>Rabbit Polyclonal anti-GFP (used for TRAP), Abcam, Cat# ab290;<br>Donkey anti Rabbit IgG (H+L) Highly Cross Adsorbed Secondary Antibody, Alexa Fluor 647 (used for flow cytometry and immunohistochemistry), Thermo Scientific, Cat# A-31573;<br>Rabbit Monoclonal Cyclophilin B (D1V5J) (western blot), Cell Signaling Technology, Cat# 43603S;<br>Donkey Monoclonal anti-rabbit IgG, horseradish (western blot), Cytiva, Cat# NA934
Validation	Antibodies were validated by the corresponding manufacturer. Rabbit Monoclonal IRF3 antibody was additionally validated using in vivo IRF3 knockout mice (Figure 4B), IRF3 gain-of-function (Figure S4H).<br>IRF3 https://www.cellsignal.com/products/primary-antibodies/irf-3-d83b9-rabbit-mab/4302<br>Phospho-Stat3 (Tyr705): https://www.cellsignal.com/products/primary-antibodies/phospho-stat3-tyr705-d3a7-xp-rabbit-mab/9145<br>Rabbit Polyclonal anti-GFP (used for TRAP): https://www.abcam.com/products/primary-antibodies/gfp-antibody-ab290.html<br>https://www.thermofisher.com/antibody/product/Donkey-anti-Rabbit-IgG-H-L-Highly-Cross-Adsorbed-Secondary-Antibody-Polyclonal/A-31573?gclid=CjwKCAjw5v2wBhBrEiwAXDDoJeAx5Sd65aJfd0Ot7xoV6r04gcs0xfcJYW2tmmuhOhCe6BQOEGD2_RoC7QEQAvD_BwE&ef_id=CjwKCAjw5v2wBhBrEiwAXDDoJeAx5Sd65aJfd0Ot7xoV6r04gcs0xfcJYW2tmmuhOhCe6BQOEGD2_RoC7QEQAvD_BwE:G:s&s_kwcid=AL!3652!3!516608152221!!!g!!!12825517856!122158235235&cid=bid_pca_aus_r01_co_cp1359_pjt0000_bid00000_0se_gaw_dy_pur_con&gad_source=1<br>Cyclophilin B (D1V5J): https://www.cellsignal.com/products/primary-antibodies/cyclophilin-b-d1v5j-rabbit-mab/43603<br>anti-rabbit IgG, horseradish: https://d3.cytivalifesciences.com/prod/COFA/NA934-1ML%20lot%204629890.pdf?

_gl=1*3swf80*_gcl_au*MTkyNTUxNzg0MC4xNzEzNDA5MTQ4

Eukaryotic cell lines

Policy information about cell lines and Sex and Gender in Research

Cell line source(s)	GT1-7 cells were gifted by Young-bum Kim (Huang et al., 2012).
Authentication	LepR transfected GT1-7 cells were determined to leptin-responsive by induction of p-STAT3 (Figure S4G).
Mycoplasma contamination	Cell lines were not tested for mycoplasma contamination.
Commonly misidentified lines (See ICLAC register)	None.

Animals and other research organisms

Policy information about studies involving animals; ARRIVE guidelines recommended for reporting animal research, and Sex and Gender in Research

Laboratory animals

Generation of mice:
IRF3-2D mice and IRF3 floxed mouse founder lines were generated as previously described (Yan et al., 2021). For loss-of-function studies, we crossed Irf3flox mice with AgRP-IRES-Cre mice to generate AgRP neuron-deficient IRF3 mice (AgI3KO). For gain-of-function studies, we 21 crossed IRF32D mice with AgRP-IRES-Cre mice to generate mice expressing constitutively active IRF3 in their AgRP neurons (AgI3-2D). We crossed transgenic Nuclear tagging and Translating Ribosome Affinity Purification (NuTRAP) mice with AgRP-IRES-Cre mice to generate the NuTRAPAgRP mouse line, from which we could isolate AgRP neuron-specific mRNA and nuclei.

Other sources:
Global IRF3 knockout (IRF3KO) mice were obtained from the RIKEN BRC Experimental Animal Division (Sato et al., 2000). C57BL/6J (WT), AgRP-IRES-Cre, POMC-IRES-Cre and B6;129S6- Gt(ROSA)26Sortm2(CAG-NuTRAP)Evdr/J (NuTRAP) mice were ordered from the Jackson Laboratory.

Animals: standard fed, fasted, and leptin-treated comparison:
6-to-11-week-old male C57BL/6J NuTRAPAgRP male mice were fed a standard chow diet ad libitum. At least one day before the experiment, mice were singly-housed in a cage with wood chip bedding and handled by the experimenter using a cupping method shown to reduce anxiety in mice (Hurst and West, 2010). Mice were either maintained on their chow diet (fed mice), or fasted (fasted mice) overnight for 18-20 hours. At zeitgeber ZT time 2-4, mice were intraperitoneally (i.p.) injected with either vehicle (PBS) or leptin (5mg/kg) and euthanized 3 hours later.
Male mice between 6-to-11 weeks of age were used for the flow cytometry experiments.
Mice between 6-to-11 weeks of age were used for all food intake behavioral experiments.
Mice between 17-to-22 weeks of age were used for telemetry experiments.
Age-matched littermates were used as controls for all experiments.

Wild animals	None.
Reporting on sex	We have made it clear throughout the manuscript that all experiments were conducted using male mice and have acknowledged the limitations of this approach in our discussion.
Field-collected samples	NA; our study did not involve field-collected samples.
Ethics oversight	All animal experiments were performed with approval from the Institutional Animal Care and Use Committees of The Harvard Center for Comparative Medicine and Beth Israel Deaconess Medical Center (IACUC protocol numbers 056-2017, 024-2020, 018-2023)

Note that full information on the approval of the study protocol must also be provided in the manuscript.

Plants

Seed stocks	NA.
Novel plant genotypes	NA.
Authentication	NA.

Flow Cytometry

Plots

Confirm that:

☒ The axis labels state the marker and fluorochrome used (e.g. CD4-FITC).

☒ The axis scales are clearly visible. Include numbers along axes only for bottom left plot of group (a 'group' is an analysis of identical markers).

☒ All plots are contour plots with outliers or pseudocolor plots.

☒ A numerical value for number of cells or percentage (with statistics) is provided.

Methodology

Sample preparation	Fluorescence Assisted nuclei sorting (FANS) for ATAC-seq: AgRP neuronal nuclei were isolated as previously described (Roh et al., 2017), with minor alterations. Briefly, dissected ARCs from 6-11-week-old mice were snap frozen and stored at - 80C. Isolated ARCs were dounce homogenized in nuclear preparation buffer (NPB; 10 mM HEPES [pH 7.5], 1.5mM MgCl2, 10 mM KCl, 250 mM sucrose, 0.1% NP-40, and 0.2 mM DTT), and homogenates were filtered through a 100 µM strainer and centrifuged to pellet the nuclei. Nuclei were washed with NPB, re-suspended in nuclear sorting buffer (10 mM Tris [pH 7.5], 40 mM NaCl, 90 mM KCl, 2 mM EDTA, 0.5 mM EGTA, 0.1% NP-40, 0.2 mM DTT), and filtered again through a 40 µM strainer. Isolated nuclei were gated by FSC, SSC and GFP-FITC fluorescence expressed in the nucleoli. With this approach we routinely obtain ~3000 AgRP neuronal nuclei per mouse. Nuclei were collected into 500-750uL PBS (0.1% NP40) in 1.5 mL microcentrifuge tubes and stored on ice.<br><br>In vivo flow cytometry: 6-to-11-week-old male C57BL/6J male mice were maintained on a standard chow diet ad libitum. Mice were handled for 5-minutes on the days prior to the experiment. Mice were fasted overnight for 19-hours, before being injected I.P. with leptin (5 mg/kg). Using a modified version of the FAST-FIN protocol51, 5-hours later, mice were rapidly perfused with 10% formalin for 1-minute, and whole hypothalami were isolated and snap-frozen in a microcentrifuge tube on dry ice. Whole mouse hypothalamus was rapidly isolated 5-hours later. Later, individual mouse hypothalami were subjected to dounce homogenization and immediately fixed using 1% formaldehyde for 7-minutes at RT, followed by quenching with 125mM glycine for 5-minutes. Nuclei were collected and resuspended in 22% OptiPrep which was layered on top of a 43% Optiprep, and centrifuged at 10,000 g for 30-minutes. Nuclei were resuspended with Blocking Buffer (without Triton-X, in PBS) and centrifuged for 10,000g for 10 min at 4C. Supernatant was removed and replaced with 500 ul premade Blocking Buffer with Triton-X in PBS and nuclei were blocked for 10 min at RT. Antibody was added (IRF3, 1:250; pSTAT3, 1:250) and incubated for 1-hour. Nuclei were washed with 1X wash buffer with triton-X, resuspended in Blocking Buffer with triton-X, and incubated with 1:500 secondary antibody (Alexa Fluor 647 Donkey anti-rabbit IgG) for 30-minutes at RT while light protected. Nuclei were then spun down (600 g for 3-minutes at 4°C) and gently resuspended in 500 ul FANS buffer with 1ul Hoechst, while in a 5 mL round-bottom FACS tube and ran on the CytoFLEX LX.
Instrument	BD FACS Aria II (FANS); CytoFLEX LX (flow cytometry).
Software	Built-in BD FACS Aria II software was used for data analysis of FANS. FLowjo software was used be data analysis of flow cytometry data.
Cell population abundance	For ATAC-seq, 10,648 +/- 1489 nuclei, from 4 mice, were pooled for each condition (fed, fasted, leptin-treated) before being subjected to ATAC-seq. For flowcytometry, with leptin-treatment ~7.76% of the parent population of a single whole-hypothalamus nuclei exhibited pSTAT3 (surrogate marker of leptin signaling), compared to ~2.95% with vehicle treatment; indicating that 4.81% (7.76 - 2.95) of cells/nuclei were leptin responsive. Next, with leptin-treatment 0.80% of the parent population of nuclei exhibit a IRF3 signal, compared to 0.42% with vehicle treatment; indicating that 0.38% of whole hypothalamus cells responded to leptin-treatment with IRF3 activation. With approximately 8% (0.38 divided by 4.81) of the cells in the hypothalamus that respond to leptin (pSTAT induction) at 5 hours also exhibiting nuclear IRF3 (we concede that we do not know for sure that these two cell populations overlap, but the ubiquity of STAT3 expression suggests this is a reasonable assumption), along with our unpublished data demonstrating that 7.59% of LepR-Cre+ cells in the hypothalamus express AgRP, our cell population abundance data appear to conform to expectations.
Gating strategy	For NuTRAP ATAC-seq experiments, isolated nuclei were gated by FSC, SSC and GFP fluorescence expressed in the nucleoli. For flow cytometry experiments, particles smaller than nuclei (black dots) were eliminated with an area plot of forward-scatter (FSC-A) versus side-scatter (SSC-A), with gating for nuclei-sized particles inside the gate (box). 2N Hoechst 33342 stained nuclei were positively gated to avoid nuclei doublets. Plots of forward-scatter (FSC) width versus FSC height were used to further exclude aggregates of two or more nuclei.

☒ Tick this box to confirm that a figure exemplifying the gating strategy is provided in the Supplementary Information.
